# Efficacy analysis of intraoperative radiotherapy in patients with early-stage breast cancer

**DOI:** 10.1186/s12935-020-01533-z

**Published:** 2020-09-11

**Authors:** Lin Wang, Minmin Zhu, Yuelong Cui, Xudong Zhang, Guowen Li

**Affiliations:** 1 Radiotherapy Inpatient Ward II, The First Affiliated Hospital of Zhengzhou University, No. 1 Eastern Jianshe Road, Zhengzhou, 450000 Henan China; 2 Nanshi Hospital of Nanyang, Nanyang, Henan China; 3 Anyang District Hospital of Puyang, Anyang, Henan China

**Keywords:** Early-stage breast cancer, Breast-conserving surgery, Intraoperative radiotherapy, Influencing factors

## Abstract

**Background:**

To analyze the clinical efficacy of intraoperative radiotherapy (IORT) after breast-conserving surgery (BCS) in patients with early-stage breast cancer (BC), and to investigate the relationship between its influencing factors and clinical efficacy and prognosis.

**Methods:**

A total of 73 patients with early-stage BC who underwent IORT after BCS in our hospital were selected in this research.

**Results:**

Kaplan-Meier survival analysis was used to analyze the related factors of BCS and IORT of disease-free survival (DFS) and overall survival (OS). It was found that only age (χ^2^ = 14.035, P = 0.003) was statistically positively correlated with the patient’s DFS, and local recurrence and metastasis rate and mortality were higher in patients over 70 years old. Log rank test was used to analyze multiple factors. Only the diameter of the applicator (χ^2^ = 70.378, P < 0.05) was statistically significant with wound complications, and the larger the diameter, the higher incidence of wound complications. The remaining risk factors did not increase the incidence of wound complications. COX multivariate analysis showed that age was an independent risk factor for DFS rate and the risk factor had no significant effect on the OS rate of patients undergoing IORT after BCS.

**Conclusions:**

IORT may be a safe form of treatment for the selected patients with early-stage BC, and can achieve satisfactory esthetic effect. Larger applicator diameters may increase the incidence of wound complications. Age is an independent risk factor for DFS in early-stage BC patients undergoing IORT after BCS.

## Background

The incidence of breast cancer (BC) has been increasing in recent years. With the wide application of mammography, color ultrasound, and magnetic resonance imaging technologies, the diagnosis rate of early BC has increased significantly [1].

Due to the increasing proportion of early BC, the surgical methods of BC have also changed accordingly. Breast-conserving surgery (BCS) has become the preferred treatment for early BC at home and abroad. Radiotherapy after BCS for early BC can further improve the local control rate and reduce the risk of recurrence [2].

Postoperative radiotherapy (PORT) after BCS can be divided into whole-breast irradiation (WBI) and partial-breast irradiation (PBI). BCS combined with postoperative external beam radiotherapy (EBRT) is considered as the “gold standard” of breast conserving therapy for early BC [3, 4]. However, the “gold standard” still has certain limitations. In order to overcome these limitations, radiotherapy workers are exploring accelerated partial breast irradiation (APBI). Intraoperative radiotherapy (IORT) is one of the important methods for APBI implementation in recent years, and it refers to a method of giving a single large dose of radiation to a tumour bed during surgery for an uneradicated tumour or suspected residual tumour [5]. It can be divided into two categories, electrons intraoperative therapy (ELIOT) and targeted intraoperative radiotherapy (TARGIT), according to the rays generated by mobile linear accelerator. ELIOT uses high energy electrons for a single large dose irradiation of the tumor bed [6]. To be specific, 3–12 MeV-E electrons are used for a intraoperative single irradiation at a dose of 21 Gy. Lead shield is applied beneath the mammary gland to protect nearby normal tissues including chest wall, heart and lungs from radiation damage [7]. The duration of treatment should be determined according to the size of the tumor bed. The advantages of ELIOT include uniform distribution of radiation, high dose rate, and availability for irradiation of irregular surgical cavity. At present, this therapy is in Phase clinical trial [8]. TARGIT is mainly implemented by the Intrabeam system, which uses a spherical applicator of 1.5–5 cm in diameter as determined by the radiotherapy physician according to the size of tumor bed to deliver 50KV low-energy X-ray irradiation for IORT of early-stage BC. It has been widely used in clinic nowadays. TARGIT is subject to a number of limitations including uneven distribution of dose, low dose rate, and fast dose tapering rate. The attenuation of rays may result in insufficient therapeutic dose. But it can protect surrounding normal tissues such as lungs and heart from radiation damage better than other radiotherapies.

In this study, IORT was performed with TARGIT, and patients were given a single radiotherapy dose of 16–20Gy. The purpose of this study was to investigate the clinical efficacy and esthetic effects of early BC patients undergoing TARGIT after BCS, and to explore related factors that may affect the clinical efficacy and prognosis of patients.

## Methods

### Clinical data

A restrospective study was conducted on 73 patients with early-stage BC who received IORT after BCS in our hospital from April 2013 to May 2014. All included patients had been definitely diagnosed with early-stage (T_1−2_N_0−1_M_0_) BC by pathological and radiological examinations. The included clinical statistics were age, tumor size, pathological characteristics of tumor tissue, tumor staging, diameter of spherical applicator, irradiation dose, the combined chemotherapy and endocrine therapy (if any) and their regimens. The pathological characteristics of tumor tissues were from the hospital’s postoperative pathology reports.

### Inclusion criteria and exclusion criteria

Inclusion criteria: (1) Patients aged ≥ 40 years old; (2) patients whose diameter of tumor ≤ 2.5 cm; (3) patients who were free of multifocal cancer as determined by preoperative imaging procedures, and free of lymphadenectasis and supraclavicular/internal mammary lymphatic metastasis and distant metastasis (DM) as determined by preoperative imaging procedures and physical examinations; (4) patients who with negative results for all margins in the first fast intraoperative pathological section examination upon the removal of tumor tissues. Exclusion criteria: (1) Patients aged < 40 years old; (2) patients with positive surgical margins and diameter of breast duct carcinoma in situ ≥ 3 cm. All patients had been naive to neoadjuvant chemotherapy, endocrine therapy, and targeted therapy before BCS by the same surgeon in our hospital. This study received the support of the Ethics Committee of the First Affiliated Hospital of Zhengzhou University. All patients singed an informed consent for IORT.

### Radiotherapy equipment and treatment parameters

The IORT equipment used in this study was Zeiss INTRABEAM system, which used the X-ray source (XRS) in the applicator to deliver 50 KV low-energy X-ray each time. All applicators used were of spherical shape and a size (2.5–4 cm) determined by the size of the tumor bed. The single irradiation dose was 16–20Gy. If the number of axillary lymph node metastases ≥ 4 identified in postoperative routine pathological examination, modified radical mastectomy would be performed. For patients whose postoperative pathogical examination suggested high risk factors such as high-grade, lobular carcinoma, vascular invasion, axillary lymph nodes positive, margin positive, extensive duktalen carcinoma in situ (DCIS), additional EBRT would be administered after the IORT. Patients for whom additional EBRT was arranged would receive an irradiation dose of 45 Gy on their planning target volume (PTV), administered in 1.8 Gy fractions. After the BCS and IORT, patients with early-stage BC need to undergo conventional chemotherapy and endocrine therapy according to estrogen receptor and progesterone receptor. The chemotherapy was administered for 4–8 cycles after the IORT. The chemotherapy regimens mainly include paclitaxel-based and anthracyclines-based regimens.

### Evaluation criteria

The patients’ local recurrence rate and incidence of wound complications were evaluated by physical examination, imaging data and pathological examinations. Indexes included in follow-up statistics: local regional recurrence (LRR) rate, DM rate, disease-free survival (DFS) rate and overall survival (OS) rate. LRR refers to supraclavicular, internal mammary, and/or axillary recurrence confirmed by imaging data or biopsy pathological results. DM is defined as metastasis in affected side of breast or in any sites other than supraclavicular, internal mammary, and/or axillary areas of the affected side confirmed by imaging data or biopsy pathological results. DFS refers to the period from the end of BCS to the patient’s death as a result of the recurrence, metastasis, or progress of tumor. OS is the period from the surgery to the patient’s death for any reason or to the last follow-up time. Harris esthetic evaluation criteria was: percentage of excellent and good results = Cases with excellent results + cases with good results / Total cases × 100% [9].

### Statistical analysis

Statistical analysis was carried out with SPSS 21.0 software. The kaplan-Meier survival curve was used to conduct a univariate analysis of the clinical efficacy of IORT for early BC. Log rank test was used for comparison between groups, and Cox regression model was used for multivariate analysis. The test level α = 0.05. There was a significant difference at P < 0.05.

## Results

### Clinical efficacy of IORT in early-stage BC

The median follow-up time was 42.6 months (range: 8–54.6 months) from the start of BCS. According to follow-up combined with imaging and pathologic biopsy, four patients occurred with local recurrence (5.5%), among which one patient had ipsilateral supraclavicular metastasis (1.4%) and was administered chemotherapy and endocrine therapy, the other three patients were identified with recurrence in tumor bed (4.1%) and were subjected to radical mastectomy on the affected side. No ipsilateral axillary lymph node metastasis was found. The time to recurrence/metastasis for the four patients was 14.1, 14.4, 42.0, and 45.9 months, respectively. The median time to recurrence/metastasis was 28.2 months. Three patients died (4.1%) without DM. By the end of follow-up, the patients’ DFS was 90.4% and OS was 95.9%. Eight patients experienced wound complications (11.0%), among which three patients had breast and axillary effusion (4.1%) on the affected side, four patients had incision edema (4.1%), two patients had incision hematoma and infection (2.7%). Debridement was performed in patients with incisional infection, and the remaining patients were administered topical or oral medication to relieve symptoms.

### Esthetic effects of post-IORT

The esthetic effects of Post-IORT patients were evaluated by Harris criteria, as shown in Table 1, the excellent and good rate (%) was 84.9%.

**Table 1:** Post-IORT esthetic evaluation of breasts by Harris criteria

Excellent (%)	Good (%)	Fair (%)	Poor (%)	Excellent and good rate (%)
38 (52.1)	24 (32.9)	9 (12.3)	2 (2.7)	62 (84.9)

*IORT* intraoperative radiotherapy

### Clinical efficacy analysis of IORT in early-stage BC

**Fig. 1:**
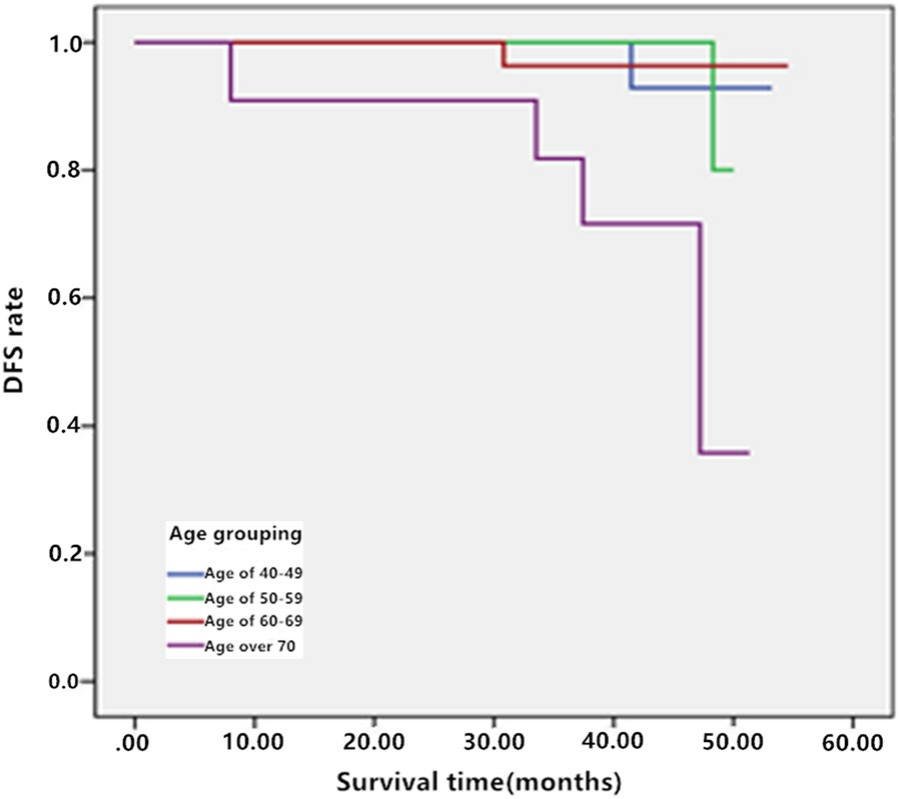
DFS curve of influence of age on patients’ survival. DFS: disease-free survival

Univariate analysis of the age factor of early-stage BC patients underwent IORT after BCS was carried out with Kaplan–Meier survival curve. The patients’ age was divided into 4 groups, group 1: age ≤ 49 years old, group 2: age between 50 and 59 years old, group 3: age between 60 and 69 years old, group 4: age ≥ 70 years old; the median survival time was 47.5 months for Group 1, 42.9 months for Group 2, 40.4 months for Group 3, and 40.3 months for Group 4. The influence of age on patients’ survival was analyzed by log rank test. The baseline characteristics of the patients and relevant univariate analysis were shown in Tables 2 and 3. The analysis of influencing factors of DFS showed that there were significant differences in DFS among different age groups (χ^2^ = 14.035, P = 0.003), and the local recurrence/metastasis rates and death rate were higher in patients ≥ 70 years old. The survival curve of the influence of age on the survival of patients was shown in Fig. 1. There was no significant difference in OS between early BC patients treated with IORT (χ^2^ = 6.933, P = 0.074). Univariate comparison and prognosis analysis such as tumor size, pathological type, histological WHO grade, applicator diameter, irradiation dose, adjuvant chemotherapy regimen and adjuvant endocrine therapy regimen were also carried out with Kaplan–Meier evaluator. The results showed that none of these factors was associated with the DFS and/or OS in early-stage BC patients receiving IORT. An analysis of the influencing factors on wound complications showed that there was significant difference in the influence of applicator diameter on the patients’ wound complications (χ^2^ = 70.378, P < 0.05), while the rest risk factors didn’t increase the incidence of wound complications. Multivariate analysis of the studied risk factors’ influence on the patients’ prognosis and survival were carried out by stepwise COX regression and the results were inverted to reveal that age was an independent risk factor for the DFS of early-stage BC patients who received IORT, while the rest risk factors had no significant effect on OS, as shown in Table 4.

**Table 2:** Basic information of the patients

Items	Number of cases (%)
Age (years)	
Group 1: 40–49	15 (20.5)
Group 2: 50–59	20 (27.4)
Group 3: 60–69	27 (37.0)
Group 4: ≥ 70	11 (15.1)
Diameter of tumor (cm)	
Group 1: < 2	26 (35.6)
Group 2: ≥ 2	47 (64.4)
Sentinel node state	
Group 1: Negative	71 (97.3)
Group 2: Positive	2 (2.7)
Postoperative pathological type	
Group 1: Intraductal carcinoma	7 (9.6)
Group 2: Infiltrating ductal carcinoma	65 (89.0)
Group 3: Others	1 (1.4)
ER State	
Group 1: Positive	59 (80.8)
Group 2: Negative	14 (19.2)
PR State	
Group 1: Positive	55 (75.3)
Group 2: Negative	14 (19.2)
Diameter of applicator (cm)	
Group 1: 2.5	5 (6.8)
Group 2: 3	36 (49.3)
Group 3: 3.5	2 (2.7)
Group 4: 4	1 (1.4)
Irradiation dose (Gy)	
Group 1: 16	2 (2.7)
Group 2: 20	71 (97.3)
Histological WHO Grade	
Group 1: Grade I–II (including grade II)	11 (15.1)
Group 2: Above grade II	62 (85.0)
Chemotherapy regimen	
Group 1: Not administered	20 (27.4)
Group 2: Mainly paclitaxel	20 (27.4)
Group 3: Mainly anthracyclines	22 (30.1)
Group 4: Others	11 (15.1)
Endocrine therapy regimen	
Group 1: Tamoxifen	9 (12.3)
Group 2: Anastrozole	28 (38.4)
Group 3: Exemestane	10 (13.7)
Group 4: Not administered	26 (35.6)

*ER* estrogen receptor, *PR* progesterone receptor

**Table 3:** Univariate analysis of IORT

Risk factors	DFS		OS		Wound complications	
	χ^2^	P	χ^2^	P	χ^2^	P
Age	14.035	0.003	6.933	0.074	0.290	0.962
Diameter of tumor	0.216	0.642	0.008	0.930	0.010	0.921
Sentinel node state	0.089	0.765	0.072	0.788	0.063	0.801
Postoperative pathological type	0.099	0.549	0.952	0.832	1.066	0.587
ER state	0.226	0.635	0.363	0.547	0.663	0.416
PR state	1.385	0.429	0.512	0.731	0.013	0.908
Diameter of applicator	1.424	0.700	4.386	0.223	70.378	0.000
Histological WHO Grade	0.094	0.759	0.760	0.383	0.051	0.822
Chemotherapy regimen	1.784	0.626	2.601	0.457	2.202	0.532
Endocrine therapy regimen	2.118	0.548	1.660	0.646	3.379	0.337

*IORT* intraoperative radiotherapy, *ER* estrogen receptor, *PR* progesterone receptor, *DFS* disease-free survival, *OS* overall survival

**Table 4:** Multivariate analysis of DFS by COX regression

	B	SE	Wald	df	Sig.	Exp (B)	The 95% CI used for Exp (B)
Age	0.174	0.072	5.767	1	0.016	1.190	1.032–1.371

*DFS* disease-free survival

## Discussion

With the discovery and application of relevant serum and tissue biomarkers and the advancement of medical technology in the diagnosis of BC, the detection rate of early BC is continually improved. Against this backdrop, the treatment options for BC are also changing and currently BCS have become the generally accepted and preferred mode of treatment for early-stage BC patients in the world as a result of the growing number of young patients. PORT also plays an important role in early-stage BC patients who want to conserve their breasts. PORT can further improve the local control rate of BC and reduce the disease’s recurrence rate by 50% [10]. Postoperative WBI, however, suffers from a number of limitations including significant adverse reactions, long treatment duration, and inconvenience for patients and their families. As a result of the in-depth studies of post-BCS recurrence modes, APBI comes into being at the opportune moment, and APBI is characterized by big fraction dose [11]. Compared to conventional fractionated radiotherapy, APBI can achieve equivalent total biological dose with smaller total irradiation dose and shorter total treatment duration. Therefore, it has become a hot research in recent studies of post-BCS in early-stage BC patients, and its clinical efficacy has been confirmed by more and more clinical studies [12].

APBI can be implemented by mammosite brachytherapy (MSB) and intracavity brachytherapy (IBT), IORT and EBRT. There are more and more clinical studies on and applications of IORT because it has the following advantages: (1) IORT is one-off and it can significantly reduced total treatment time; (2) IORT is administered on the tumor bed under direct vision and therefore capable of demarcating the target tumor bed area more precisely and eliminating the uncertainties of target area during post-WBI SIB; (3) IORT intraoperatively delivers irradiation to the tumor bed for reducing the risk of fast proliferation of postoperative residual tumor cells [13]; (4) IORT can greatly protect the surrounding normal tissues such as lungs and mammary gland from radiation damage for its fast dose tapering rate; (5) IORT may change the microenvironment of tumor for the control of the proliferation and differentiation of residual tumor cells on tumor bed and control local recurrence [14]. The application of APBI has been listed as an option for the treatment of early-stage BC patients in NCCN Guidelines [15]. Therefore, more and more in-depth researches on alternative treatment models were carried out. In early-stage BC patients who underwent BCS followed by routine WBI or no radiotherapy, 60–80% of the local recurrences of tumor are in the tumor bed and its surrounding tissues and only 0.6–5% of the patients have recurrences farther away from the tumor bed [16]. Multi-center randomized controlled studies compared the efficacy and safety of APBI and WBI concluded that APBI was effective and safe in the treatment of selected patients [17, 18]. As one of implementation methods of APBI, IORT is a highly effective topical therapy administered intraoperatively after the surgical removal of malignant tumor tissue. It delivers a one-off big dose of irradiation to tumor bed in order to kill tumor cells and avoid causing damage to surrounding normal health tissues outside the range of exposures. Lemanski et al. [19] reported that IORT for selected BC patients showing low recurrence rate, good esthetic effect and excellent satisfaction.

Grobmyer et al. [20] conducted a retrospective study on patients who received treatment with Intrabeam system. After 1 year of follow-up, 92% of the patients were satisfied by the esthetic appearance of their breasts and none had experienced local recurrence. A study by Piotrowski et al. suggested that a single IORT could be used as an alternative for 6–7 weeks of PORT [21]. Jacobs et al. [22] conducted a study on the toxic reactions of IORT and concluded that, just like EBRT, IORT had toxic reactions tolerable by the patients. Moreover, a recent clinical trial entitled TARGIT-R showed that this therapy had a low wound complications incidence and local recurrence rate [23]. The above-mentioned studies and our retrospective study showed the clinical efficacy and esthetic effects of IORT in early-stage BC patients were satisfactory. Therefore, IORT deserves clinical application and promotion.

However, there are also some shortcomings for IORT: it may extend the operation time and defer wound healing, it has to be done intraoperatively before postoperative pathological report is available, and it is liable to the the influence of a number of factors which may have adverse impact on clinical therapeutic effect, including oversize surgical cavity, short exposure duration, a shorter-than-safe-distance between the incision margin and chest skin, and inconvenient operative dissection conditions which make it hard to avoid the escape of some planned target areas from irradiation exposure [24]. There are a lot of clinical studies on the clinical efficacy and esthetic effects of IORT. However, studies for identifying the risk factors that may affect the wound complication incidence and local recurrence rate are relatively fewer. This retrospective study proposed that patients’ age was an independent risk factor for the DFS of patients underwent IORT. Patients aged 70 or above may have higher local recurrence rate, metastisis rate and mortality rate than other patients. This is consistent with the results of relevant study [25]. During our analysis, it was found that large applicator diameter was significantly associated with the increase of wound complications. This finding is consistent with the results of Rakhra et al. [26]. However, it cannot be ruled out that this effect is due to large incisions or uneven distribution of exposure doses. Although most studies show that TARGIT is safe, there is a study suggesting that the local recurrence rate is still a bit high over a long follow-up period after TARGIT [27]. In addition, TARGIT has a high possibility of fat liquefaction and necrosis in some surgical sites, which deserves our attention and consideration.

## Conclusions

This retrospective study showed that it was safe for early-stage BC patients to receive IORT and that IORT was associated with low incidence of toxic reactions and satisfactory clinical efficacy. In spite of many limitations including its relatively small sample size, short follow-up time, and the limited availability of clinical studies on the influencing factors of therapeutic effects, certain safety and efficacy concerns relating to IORT had been identified, which requires us to give due considerations to patients’ age when selecting eligible patients and demands our prudence when selecting diameter of spherical applicator for IORT. With the development and progress of technology, people will attach more importance and pay more attention to clinical trials with large sample size and long follow-up time, and the application of IORT will be more and more extensive.

## Abbreviations

IORT: Intraoperative radiotherapy
BCS: Breast-conserving surgery
BC: Breast cancer
DFS: Disease-free survival
OS: Overall survival
WBI: Whole-breast irradiation
PBI: Partial-breast irradiation
EBRT: External beam radiotherapy
SIB: Simultaneous integrated boost
APBI: Accelerated partial breast irradiation
XRS: X-ray source
ELIOT: Electrons intraoperative therapy
TARGIT: Targeted intraoperative radiotherapy
DM: Distant metastasis
DCIS: Duktalen carcinoma in situ
PTV: Planning target volume
LRR: Local regional recurrence
DFS: Disease-free survival
PORT: Postoperative radiotherapy
MSB: Mammosite brachytherapy
IBT: Intracavity brachytherapy

## Availability of data and materials

All data generated or analysed during this study are included in this published article.

## References

[1] Chen WQ, Zheng RS (2015). Incidence, mortality and survival analysis of breast cancer in China. Chin J Clin Oncol.

[2] Li YH, Zhang LH, Chen X (2018). Research progress of breast-conserving surgery combined with intraoperative radiotherapy for early breast cancer. Chin J Endocr Surg.

[3] Reintgen C, Reintgen D, Solin LJ (2010). Advances in local regional treatment for patients with early target breast cancer: a review of the field. Clinic Breast Cancer.

[4] Noël G, Mazeron JJ (2001). Favourable and unfavourable effects on long-term survival of radiotherapy for early breast cancer: an overview of the randomised trials. Cancer Radiather.

[5] Reitsamer R, Sedlmayer F, Kopp M, Kametriser G, Menzel C, Glueck S (2008). Concepts and techniques of intraoperative radiotherapy (IORT) for breast cancer. Breast Cancer.

[6] Leonardi MC, Maisonneuve P, Mastropasqua MG, Cattani F, Fanetti G, Morra A (2017). Comparison of treatment outcome between invasive lobular and ductal carcinomas in patients receiving partial breast irradiation with intraoperative electrons. Int J Radiat Oncol Biol Phys.

[7] Fastner G, Reitsamer R, Sedlmayer F (2014). Partial breast irradiation with intraoperative electrons versus conventional external whole breast irradiation for early breast cancer. Results of the ELIOT trial. Strahlenther Onkol.

[8] Livi L, Meattini I, Marrazzo L, Simontacchi G, Pallotta S, Saieva C (2015). Accelerated partial breast irradiation using intensity-modulated radiotherapy versus whole breast irradiation: 5-year survival analysis of a phase 3 randomised controlled trial. Eur J Cancer.

[9] Yin WB, Gu XZ (2002). Radiation oncology.

[10] Darby S, McGale P, Correa C, Taylor C, Arriagada R, Early Breast Cancer Trialists’ Collaborative Group (EBCTCG) (2011). Effect of radiotherapy after breast-conserving surgery on 10-year recurrence and 15-year breast cancer death: meta-analysis of individual patient data for 10,801 women in 17 randomised trials. Lancet.

[11] Correa C, Harris EE, Leonardi MC, Smith BD, Taghian AG, Thompson AM (2017). Accelerated partial breast irradiation: executive summary for the update of an ASTRO evidence-based consensus statement. Pract Radiat Oncol.

[12] Wickberg A, Holmberg L, Adami HO, Magnuson A, Villman K, Liljegren G (2014). Sector resection with or without postoperative radiotherapy for stage I breast cancer: 20⁃year results of a randomized trial. J Clin Oncol.

[13] Williams NR, Pigott KH, Brew-Graves C, Keshtgar MR (2014). Intraoperative radiotherapy for breast cancer. Gland Surg.

[14] Holmes DR (2014). Intraoperative radiotherapy in breast conserving surgery. J Surg Oncol.

[15] Wallace M, Ye H, Wobb J, Jawad MS, Dekhne N, Brabbins DS (2015). Accelerated partial-breast irradiation (APBI) versus whole-breast irradiation (WBI) in treatment of the biological subtypes of breast cancer:an analysis of comparative effectiveness. J Clin Oncol.

[16] Subhedar P, Olcese C, Patil S, Morrow M, Van Zee KJ (2015). Decreasing recurrence rates for ductal carcinoma in situ: analysis of 2996 women treated with breast⁃conserving surgery over 30 years. Ann Surg Oncol.

[17] Meattini I, Saieva C, Miccinesi G, Desideri I, Francolini G, Scotti V (2017). Accelerated partial breast irradiation using intensity modulated radiotherapy versus whole breast irradiation: health-related quality of life final analysis from the Florence phase 3 trial. Eur J Cancer.

[18] Harat A, Harat M, Makarewicz R (2016). Whole breast irradiation vs. APBI using multicatheter brachytherapy in early breast cancer-simulation of treatment costs based on phase 3 trial data. J Contemp Brachyther.

[19] Lemanski C, Bourgier C, Draghici R, Thezenas S, Morel A, Rouanet P (2020). Intraoperative partial irradiation for highly selected patients with breast cancer: Results of the INTRAOBS prospective study. Cancer Radiother.

[20] Grobmyer SR, Lightsey JL, Bryant CM, Shaw C, Yeung A, Bhandare N (2013). Low-kilovoltage, single-dose intraoperative radiation therapy for breast cancer:resuits and impact on a multidisciplinary breast cancer program. J Am Coll Surg..

[21] Piotrowski I, Kulcenty K, Wichtowski M, Murawa D, Suchorska W (2017). Intraoperative radiotherapy of breast cancer and its biological effects. Breast Care.

[22] Jacobs DHM, Speijer G, Petoukhova AL, Roeloffzen EMA, Straver M, Marinelli A (2018). Acute toxicity of intraoperative radiotherapy and external beam-accelerated partial breast irradiation in elderly breast cancer patients. Breast Cancer Res Treat.

[23] Valente SA, Tendulkar RD, Cherian S, O’Rourke C, Greif JM, Bailey L (2016). TARGIT-R (Retrospective): North American experience with intraoperative radiation using low-kilovoltage X-rays for breast cancer. Ann Surg Oncol.

[24] Hanna GG, Kirby AM (2015). Intraoperative radiotherapy in early stage breast cancer: potential indications and evidence to date. Br J Radiol.

[25] Zeidan YH, Habib JG, Ameye L, Paesmans M, de Azambuja E, Gelber RD (2018). Postmastectomy radiation therapy in women with T1⁃T2 tumors and 1 to 3 positive lymph nodes: analysis of the breast international group 02⁃98 trial. Int J Radiat Oncol Biol Phys.

[26] Rakhra S, Bethke K, Strauss J, Hayes JP, Hansen N, Khan SA (2017). Risk factors leading to complications in early-stage breast cancer following breast-conserving surgery and intraoperative radiotherapy. Ann Surg Oncol.

[27] Wenz F, Welzel G, Blank E, Hermann B, Steil V, Sütterlin M (2010). Intraoperative radiotherapy as a boost during breast-conserving surgery using low-kilovoltage X-rays: the first 5 years of experience with a novel approach. Int J Radiat Oncol Biol Phys.

